# Reliability and validity of ADHD diagnostic criteria in the Assessment System for Individuals with ADHD (ASIA): a Japanese semi-structured diagnostic interview

**DOI:** 10.1186/s12888-015-0525-7

**Published:** 2015-06-20

**Authors:** Toshinobu Takeda, Yui Tsuji, Teruhisa Uwatoko, Hiroshi Kurita

**Affiliations:** Department of Clinical Psychology, 125-1 Daiku-cho, Oomiya-higashihairu, Shichijyo-dori, Shimogyo-ku, Kyoto, 600-8268 Japan; Ryukoku University Health Care Center, Kyoto, Japan; Kyoto University Health Care Service, Kyoto, Japan; Zenkoku Ryoiku-Sodan Center, Tokyo, Japan

**Keywords:** Adults, Attention-deficit/hyperactivity disorder, Diagnosis, Psychometric properties, Semi-structured diagnostic interview

## Abstract

**Background:**

With reports of a high prevalence of attention-deficit/hyperactivity disorder (ADHD) in adults, publication of ADHD diagnostic criteria in DSM-5, and the urgent need for a relevant diagnostic instrument conforming to DSM-5, we developed the Assessment System for Individuals with ADHD (ASIA), a Japanese semi-structured diagnostic interview. We report here the reliability and validity of ASIA ADHD diagnostic criteria.

**Methods:**

ASIA ADHD criterion A corresponds to DSM-5 ADHD criterion A and has 144 original questions assessing nine inattention symptoms and nine hyperactivity–impulsivity symptoms, each having four childhood and four adulthood questions. The 144 questions are evaluated on a 3-point frequency scale. ASIA ADHD criteria B to E correspond to DSM-5 ADHD criteria B to E and are evaluated on a 2-point scale. ASIA was administered to 60 adults (mean age, 29.9 ± 9.0 years; 28 males; 36 ADHD and 24 non-ADHD participants diagnosed by consensus of two experts).

**Results:**

For ASIA ADHD criterion A, values of Cronbach’s α for the adulthood and childhood inattention and hyperactivity–impulsivity symptoms ranged from 0.64 to 0.90. Values of κ for two independent raters ranged from 0.98 to 1.00 for the 144 questions and raw agreement rates ranged from 0.97 to 1.00 for criteria B, C, D, and E. The consensus DSM-5 diagnoses endorsed 59 of the 60 ASIA diagnoses (ADHD and non-ADHD). The ADHD group scored significantly higher on 125 of the 144 questions for criterion A than the non-ADHD group. Correlations between ASIA total and subscale scores in adulthood and corresponding scores on the Japanese version of the Conners’ Adult ADHD Scales-Self Report were high.

**Conclusions:**

ASIA ADHD criteria showed acceptable psychometric properties, although further investigation is necessary. The use of ASIA ADHD criteria could facilitate clinical practice and research into adult ADHD in Japan.

**Electronic supplementary material:**

The online version of this article (doi:10.1186/s12888-015-0525-7) contains supplementary material, which is available to authorized users.

## Background

There is a high prevalence worldwide of attention-deficit/hyperactivity disorder (ADHD) in adults, children, and adolescents. ADHD symptoms interfere with various aspects of daily life, such as education, work, and social relationships. Current estimates of the prevalence of adult ADHD are as high as 3.4 % (1.2–7.3 %) [1], indicating a substantial need for adult ADHD therapy in many countries.

A relevant diagnosis is imperative for the administration of appropriate interventions for psychiatric or psychological problems. However, there are considerable difficulties in diagnosing adult ADHD, mainly because of high comorbidity rates and uncertainty in the recall of childhood symptoms by potential ADHD patients and their parents. The pattern of comorbidity of ADHD changes from childhood through adolescence [2]. After adolescence, internalizing disorders and substance use disorders dominate externalizing disorders, such as oppositional defiant disorder and conduct disorder [3]. Internalizing disorders such as depression and anxiety interfere to varying degrees with attention, which can mimic ADHD symptoms of inattention. Sometimes, irritability related to depression may cause restlessness or impulsive behaviors. Moreover, as DSM-5 specifies that ADHD symptoms should be present prior to 12 years of age [4], clinicians must seek evidence for childhood ADHD features to diagnose adult ADHD. In many cases, this evidence is based on the patient’s memory of experiences that happened more than 10 years ago. Some patients (and their parents) do possess a clear memory of their childhood and have access to additional information, such as school and medical records, examination papers, and schoolwork illustrating childhood behavior patterns, but others do not. In addition, without a systematic diagnostic interview, diagnostic procedures for adult ADHD can differ from clinician to clinician.

Since the publication of DSM-IV [5], the DSM diagnostic criteria for ADHD have become the worldwide standard. A diagnostic interview faithful to these criteria could standardize ADHD diagnosis and improve both interventions for ADHD and research in the field. There are a few semi-structured diagnostic interviews for adult ADHD (i.e., Conners’ Adult ADHD Diagnostic Interview for DSM-IV [CAADID] [6], the Diagnostisch Interview voor ADHD [the Diagnostic Interview for ADHD in Adults, DIVA] [7] and the Adult ADHD Investigator Symptom Rating Scale [AISRS] [8]). However, all these interviews have been developed in Western countries according to DSM-IV criteria. Ideally, a semi-structured diagnostic interview should be developed with due consideration of the cultural aspects of the country in which it is to be used, partly because the interpretation of behavior as “normal” or “abnormal” is based on knowledge of the group’s cultural behavioral norms [9].

DSM-5 provides more example symptoms for adult (age ≥17) ADHD than DSM-IV. To our knowledge, there is no diagnostic interview for adult ADHD based on the DSM-5 criteria. Even if such an interview is developed in the near future in Western countries, it may not be readily available in Japan and such an instrument may not be culturally valid in Japan. Therefore, we created the Assessment System for Individuals with ADHD (ASIA) for adult ADHD, a semi-structured interview based on DSM-5 to help Japanese professionals diagnose adult ADHD in a standardized way. The objective of this study was to test the reliability and validity of ADHD diagnostic criteria in the ASIA for adults with and without ADHD.

## Methods

### Instrument: ASIA

ASIA is a 56-page Japanese instrument consisting of three parts: (1) the semi-structured diagnostic interview for adults (age ≥17) with possible ADHD, (2) a form recording additional participant information, (3) a rating sheet, and (4) a diagnostic algorithm (professionals can request more information about ASIA from the first author).

#### Part one

Part one, the semi-structured ADHD diagnostic interview for which we report reliability and validity in this paper, contains ADHD criteria (A to E) similar to those in DSM-5 as well as questions devised by the authors to quantify in detail the criteria. Criterion A, the most important of the ASIA ADHD criteria, consists of nine inattention symptoms and nine hyperactivity–impulsivity symptoms corresponding to the inattention and hyperactivity–impulsivity symptoms in DSM-5 ADHD criterion A. Each ASIA ADHD criterion A symptom has four questions evaluating present behavior (the average state for the last 6 months) in adulthood (age ≥17) and four questions evaluating childhood behavior (the most prominent state before age 12) on a 3-point scale (0 = never, 1 = sometimes, or 2 = often/always [often or more]). Of the four questions for each symptom, the first question most faithful to the DSM-5 symptom description; other three questions were devised to obtain further detail about each symptom, using relevant examples of the behavior. A response of “often/always (often or more)” to one or more questions for each symptom in childhood or adulthood constitutes a positive score for that symptom in that period. ASIA ADHD criterion A has a total of 144 questions (eight questions, four each for adulthood and childhood, for each of nine inattention and nine hyperactivity–impulsivity symptoms; see Additional file 1 for the questions). The ASIA ADHD criteria B, C, D, and E were each evaluated with a single question on a 2-point scale (0 = No, 1 = Yes).

The 144 questions for criterion A and four questions for criteria B to E were developed as follows: We created draft questions to reflect the ADHD symptoms in the proposed revision of DSM-5 in 2012 [10] based on our clinical experiences with ADHD patients and referring to existing interviews or questionnaires for child and adult ADHD (CAADID [6], DIVA [7], The Kiddie-Schedule for Affective Disorders and Schizophrenia-present state fourth version revised (K-SADS-P IVR) [11], K-SADS-Present and Lifetime Version (K-SADS-PL) [12], ADHD rating scale-IV [ADHD-RS-IV] [13], Wender Utah rating scale [WURS] [14], and Conners’ adult ADHD rating scales [CAARS] [15]). We conducted intensive reviews of the draft questions, and a pilot study of the draft ASIA ADHD criteria with a small sample of adult ADHD patients, to modify or eliminate inappropriate questions. Although ADHD criterion A symptom descriptions in the proposed revision of DSM-5 were not very different from those in DSM-5, the proposed revision of DSM-5 contained some new impulsivity symptoms. To ensure that our ADHD diagnostic criteria conformed to those in DSM-5, we removed four questions about impulsivity symptoms, producing a final version of the ASIA ADHD with 144 questions for criterion A and four questions for criteria B to E (See Additional file 1 for sample questions).

#### Part two

Part two contains 12 standardized sections that require adult respondents to provide information other than that concerning ADHD criteria (this part of the instrument was not tested for reliability and validity in this study): (1) demographic data (name, age, date of birth, gender); (2) prenatal and perinatal history; (3) neonatal history; (4) past medical history; (5) past psychiatric history; (6) current medical conditions; (7) current psychiatric comorbidities; (8) family history (of ADHD, autism spectrum disorder [ASD], learning disorders, intellectual disability, other psychiatric disorders); (9) educational history (academic background and history of drop grade); (10) job history; (11) economic status (income); and (12) collateral information (from parents or from documents such as report cards). As some questions about demographic, psychiatric, familial, or economic issues may be viewed as uncomfortable, especially to Japanese respondents, these questions were placed at the end of the interview.

An ASIA interview can last 1–2 h, which is an appropriate and feasible length of time for a personal interview [9].

#### Parts three and four

**Fig. 1:**
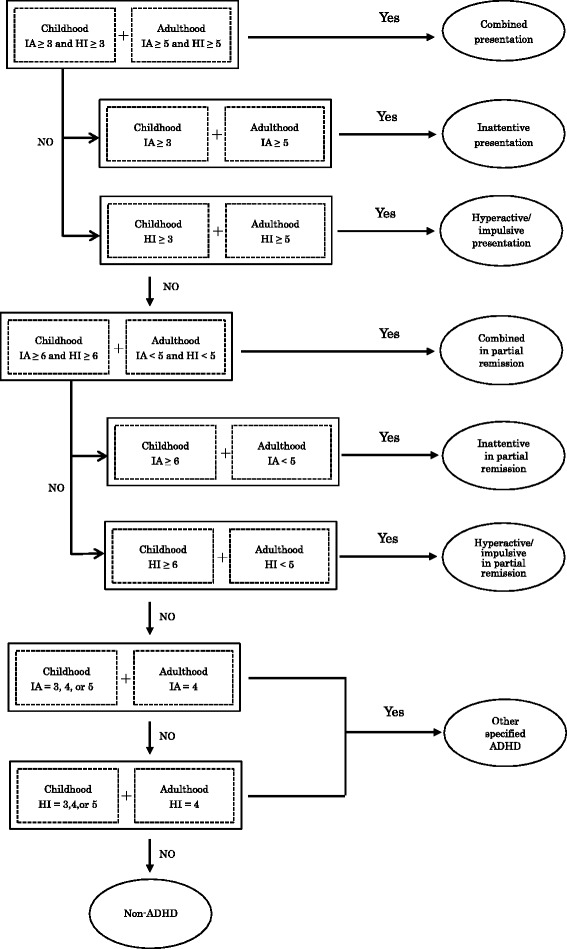
Diagnostic algorithm in the Assessment System for Individuals with ADHD (ASIA). Note: Each digit stands for the number of symptoms. IA: inattention, HI: hyperactivity/impulsivity

The interview results are recorded on the rating sheet (part three of ASIA). The ASIA ADHD diagnostic algorithm (part four, Fig. 1) draws on these ratings and guides the clinician in diagnosing ADHD on the basis of the number of positive symptoms for inattention and hyperactivity–impulsivity in childhood and adulthood. Although this diagnostic algorithm basically conforms to DSM-5, we implemented four operational rules not noted in DSM-5 to increase the efficiency of the ASIA ADHD diagnosis, as follows: (1) More than two inattention or hyperactivity–impulsivity symptoms in childhood and three to five inattention or hyperactivity-impulsivity symptoms in childhood are required for diagnosing ADHD and other specified ADHD (having symptoms characteristic of ADHD with clinically significant impairment in important areas of functioning but not meeting the full criteria for ADHD) in adults, respectively; (2) Four inattention or hyperactivity–impulsivity symptoms in adulthood are required for diagnosing other specified ADHD; (3) To fulfill criterion D (clear evidence that the symptoms interfere with or reduce the quality of social, academic, or occupational functioning), possible patients have to have a Global Assessment of Functioning score under 71; and (4) Results of the patient interview are given most weight in the diagnosis unless collateral information strongly contradicts them.

### Participants

Participants were 30 adults who had visited the psychiatric clinic in which the first author works with complaints of inattention, disorganization, hyperactivity, or impulsivity (most of whom considered themselves to have ADHD) and 30 adults who had expressed interest in participating in the research via the webpage of the peer group for adult ADHD/ASD or the first author’s webpage, most of whom considered themselves to have ADHD or another psychiatric disorder. They were recruited from May, 2013–April, 2014. Of the 60 participants (28 males; mean age = 29.9 ± 9.0 years, range = 18–49), 36 (17 males, 19 females) and 24 (11 males, 13 females) were subsequently diagnosed with ADHD or non-ADHD, respectively, using consensus diagnoses (see Procedure for detail).

Of the 36 participants with ADHD, 14 had psychiatric comorbidities (a participant can have one or more comorbidities), as follows: major depressive disorder (*n* = 5); ASD (*n* = 3); obsessive–compulsive disorder (*n* = 2); developmental coordination disorder (*n* = 2); dysthymic disorder (*n* = 1); tic disorder (*n* = 1); post-traumatic stress disorder (*n* = 1). Of the 24 participants without ADHD, four had major depressive disorder, obsessive–compulsive disorder, ASD, or bipolar II disorder.

The mean IQ of the 60 participants was 102.3 ± 12.0. The ADHD and non-ADHD groups showed no significant difference in age (ADHD, mean = 29.0 ± 8.0 years; non-ADHD, mean = 31.2 ± 10.3; *t*(58) = 0.94) and gender ratio (rate of male, ADHD, 47.2 %; non-ADHD, 44.0 %, *χ*^2^ = 0.03). The mean IQ was significantly lower in the ADHD group (mean = 99.4 ± 12.0) compared to the non-ADHD group (mean = 106.7 ± 10.9) (*t*(58) = 2.38, *p* < 0.05).

This study was approved by the ethics committee of Ryukoku University, Japan. After receiving a detailed explanation of the study from the first author, all the participants gave written informed consent to participate in the study.

### Procedure

The first author conducted the ASIA evaluation by interviewing each participant for approximately 1–1.5 h. The second author independently conducted an ASIA evaluation by watching the first author’s videotaped interview of each participant. During the face-to-face interviews, cards were used to illustrate the ASIA interview structure for the participants.

Common psychiatric comorbidities in ADHD were screened using the Japanese versions of the Beck Depression Inventory-II [16], the MINI screener [17, 18], and the Autism Spectrum Quotient [19]. If a participant obtained a positive score for any psychiatric disorder, the diagnosis was confirmed by clinical interview by the first author according to DSM-IV/DSM-5 (for ASD). Developmental coordination disorder or tic disorder were diagnosed according to DSM-IV if the clinical interview confirmed that symptoms relevant to the disorder existed and severely affected the participant’s daily life.

The consensus diagnoses of ADHD and non-ADHD were based on the DSM-5 criteria and made by experienced clinicians (third and fourth authors) using all the available participant information (except for their ASIA criteria data), including data on present behavior and symptoms on the Japanese version of Conners’ Adult ADHD Rating Scales-Self Report (J-CAARS-S) [20, 21]. Additional participant records used in the consensus diagnoses included interviews with their mothers, academic records, participants’ written answers to open-ended questions about ADHD symptoms, past medical history, past psychiatric history, and family history. The third and fourth authors made their diagnoses independently by referring to these data. If the diagnoses for a case agreed, a consensus diagnosis was recorded. If the diagnoses disagreed, this was discussed until a consensus diagnosis was reached. By consensus of the two experts, 36 and 24 participants were diagnosed with ADHD and non-ADHD, respectively. The concurrent validity and discriminant validity of the ASIA ADHD criteria were tested on the basis of the consensus diagnoses.

The first author administered the ASIA to the participants and was blind to participant information, including the J-CAARS-S scores and the additional records used in the consensus diagnoses. This author knew only that participants suspected that they suffered from, or were interested in, ADHD or other psychiatric disorders. The second author (who did not participate in the consensus diagnoses and was blind to any participant information except for the ASIA ratings that she had given) determined the ASIA ADHD diagnoses of the 60 participants on the basis of her ASIA ratings according to the algorithm (Fig. 1).

### Statistical analysis

Based on participants’ responses to a question on a 3-point frequency scale rated by the second author, values of Cronbach’s α as a measure of internal consistency reliability were calculated for the nine inattention symptoms and the nine hyperactivity–impulsivity symptoms for both adulthood and childhood. Interrater reliability (κ) was calculated for the 144 questions from data evaluated by the first and second authors. For the criteria B questions (onset prior to age 12), C (pervasiveness in setting), D (impairments), and E (not explained by another mental disorder), which used “Yes” and “No” responses, only raw agreement rates were calculated because of a lack of variance in one rater’s answers that made κ incalculable.

To examine the concurrent validity of the ASIA criteria, the agreement of the diagnoses of ADHD and its subtypes were compared for the ASIA and consensus DSM-5 diagnoses. The association between ASIA ADHD criterion A total and subscale scores in adulthood and childhood and the corresponding J-CAARS-S subscale scores was also examined as a measure of concurrent validity using the Pearson product–moment correlation coefficient (*r*). The J-CAARS-S is based on DSM-IV, but we considered its use appropriate as ADHD symptom descriptions in DSM-IV and DSM-5 are similar. To test discriminant validity of ASIA ADHD criterion A, the second authors’ ratings of the 144 questions for the ADHD and the non-ADHD groups according to the consensus diagnoses were compared using the Mann–Whitney *U* test. Statistical analyses were performed using SPSS version 22 (Armonk, NY: IBM Corp.) with the significance level set at *p* < 0.05 (two-tailed test).

## Results

### Reliability

Table 1 shows values of α for the nine adulthood and nine childhood inattention symptoms ranging from 0.74 to 0.89 and 0.75 to 0.90, respectively. Table 1 also shows values of κ for the 36 adulthood and 36 childhood inattention questions ranging from 0.98 to 1.00. Table 2 shows values of α for the nine adulthood and nine childhood hyperactivity–impulsivity symptoms ranging from 0.64 to 0.86 and 0.74 to 0.92, respectively. Table 2 also shows values of κ for the 36 adulthood and 36 childhood hyperactivity–impulsivity questions ranging from 0.98 to 1.00.

**Table 1:** Internal consistency reliability, interrater reliability, and discriminant validity for ASIA ADHD criterion A inattention symptoms

Inattention (9 symptoms: a to i)	α	κ	Mean score ± SD		*p* (*U*-test)
4 questions (1–4 each for adulthood and childhood)			ADHD (*n* = 36)	non-ADHD (*n* = 24)	
a. Careless					
adulthood (age ≥17)	0.75				
a-1, careless mistake		1.00	1.64 ± 0.49	0.96 ± 0.69	0.000
a-2, careless mistake (others)		0.98	1.42 ± 0.73	0.38 ± 0.65	0.000
a-3, use compensation strategy		1.00	1.47 ± 0.73	0.33 ± 0.65	0.000
a-4, fast and sloppy		0.98	1.93 ± 0.76	0.75 ± 0.68	0.002
childhood (age < 12)	0.75				
a-1, careless mistake		1.00	1.64 ± 0.64	0.67 ± 0.70	0.000
a-2, careless mistake (others)		1.00	1.44 ± 0.74	0.50 ± 0.72	0.000
a-3, help from others		1.00	0.67 ± 0.79	0.21 ± 0.51	0.014
a-4, fast and sloppy		1.00	1.53 ± 0.70	0.54 ± 0.51	0.000
b. Difficulty sustaining attention					
adulthood	0.81				
b-1, short attention span		1.00	1.47 ± 0.74	0.33 ± 0.57	0.000
b-2, short attention span (others)		1.00	0.81 ± 0.82	0.04 ± 0.20	0.000
b-3, space out		0.98	1.61 ± 0.76	0.42 ± 0.34	0.000
b-4, a huge gap in concentration		1.00	1.75 ± 0.68	0.50 ± 0.50	0.000
childhood	0.86				
b-1, short attention span		1.00	1.39 ± 0.72	0.25 ± 0.44	0.000
b-2, short attention span (others)		1.00	0.97 ± 0.91	0.04 ± 0.20	0.000
b-3, space out		0.98	1.69 ± 0.58	0.42 ± 058	0.000
b-4, a huge gap in concentration		1.00	1.44 ± 0.73	0.25 ± 0.61	0.000
c. Does not listen					
adulthood	0.82				
c-1, does not listen		1.00	1.44 ± 0.70	0.38 ± 0.58	0.000
c-2, does not listen (others)		1.00	0.94 ± 0.86	0.21 ± 0.42	0.000
c-3, compensation strategy		1.00	1.03 ± 0.85	0.25 ± 0.44	0.000
c-4, can not focus on conversation		1.00	1.39 ± 0.69	0.42 ± 0.50	0.000
childhood	0.81				
c-1, does not listen		0.98	1.08 ± 0.81	0.13 ± 0.34	0.000
c-2, does not listen (others)		1.00	0.89 ± 0.75	0.17 ± 0.38	0.000
c-3, compensation strategy		1.00	0.83 ± 0.84	0.21 ± 0.41	0.001
c-4, can not focus on conversation		1.00	1.33 ± 0.72	0.08 ± 0.28	0.000
d. Do not follow through					
adulthood	0.74				
d-1, does not follow through		1.00	1.22 ± 0.72	0.29 ± 0.46	0.000
d-2, does not follow through (others)		1.00	0.98 ± 0.86	0.08 ± 0.28	0.000
d-3, can not finish task		1.00	1.44 ± 0.73	0.58 ± 0.65	0.001
d-4, can not do as instructed		1.00	0.67 ± 0.76	0.13 ± 0.45	0.000
childhood	0.80				
d-1, does not follow through		1.00	1.36 ± 0.72	0.21 ± 0.42	0.000
d-2, does not follow through (others)		1.00	0.94 ± 0.79	0.21 ± 0.41	0.000
d-3, can not finish task		1.00	0.64 ± 0.76	0.25 ± 0.44	0.046
d-4, can not do as instructed		1.00	1.08 ± 0.81	0.13 ± 0.33	0.000
e. Difficulty organizing					
adulthood	0.82				
e-1, difficulty organizing		1.00	1.61 ± 0.60	0.42 ± 0.65	0.000
e-2, difficulty managing time (others)		1.00	0.75 ± 0.84	0.50 ± 0.78	n.s.
e-3, difficulty prioritizing		1.00	1.64 ± 0.59	0.38 ± 0.57	0.000
e-4, difficulty getting things done		1.00	1.31 ± 0.71	0.58 ± 0.71	0.001
childhood	0.81				
e-1, difficulty organizing		1.00	1.33 ± 0.79	0.25 ± 0.53	0.000
e-2, difficulty managing time (others)		1.00	1.08 ± 0.94	0.29 ± 0.69	0.001
e-3, difficulty prioritizing		1.00	1.44 ± 0.81	0.50 ± 0.72	0.000
e-4, difficulty getting things done		1.00	1.61 ± 0.68	0.29 ± 0.46	0.000
f. Avoid mental effort					
adulthood	0.77				
f-1, avoids mental effort		1.00	1.33 ± 0.75	0.33 ± 0.48	0.000
f-2, difficulty taking action (others)		1.00	1.03 ± 0.85	0.30 ± 0.47	0.001
f-3, puts off cumbersome things		1.00	1.42 ± 0.73	0.71 ± 0.69	0.001
f-4, procrastination		1.00	1.58 ± 0.65	0.58 ± 0.65	0.000
childhood					
f-1, avoids mental effort	0.80	1.00	1.39 ± 0.84	0.42 ± 0.50	0.000
f-2, difficulty taking action (others)		1.00	1.19 ± 0.90	0.42 ± 0.50	0.001
f-3, puts off cumbersome things		1.00	0.92 ± 0.87	0.21 ± 0.41	0.001
f-4, procrastination		1.00	0.86 ± 0.76	0.08 ± 0.40	0.000
g. Loses things					
adulthood	0.89				
g-1, loses things		1.00	1.25 ± 0.65	0.42 ± 0.77	0.000
g-2, loses things (others)		1.00	1.17 ± 0.87	0.38 ± 0.71	0.001
g-3, help from others		1.00	0.92 ± 0.81	0.17 ± 0.48	0.000
g-4, looks for things		1.00	1.50 ± 0.65	0.50 ± 0.78	0.000
childhood					
g-1, loses things	0.90	1.00	1.19 ± 0.82	0.33 ± 0.56	0.000
g-2, loses things (others)		1.00	1.14 ± 0.93	0.29 ± 0.62	0.000
g-3, help from others		0.89	1.00 ± 0.82	0.21 ± 0.50	0.000
g-4, looks for things		1.00	1.17 ± 0.77	0.21 ± 0.50	0.000
h. Easily distracted					
adulthood	0.81				
h-1, easily distracted		1.00	1.53 ± 0.65	0.58 ± 0.71	0.000
h-2, easily distracted (others)		1.00	0.81 ± 0.89	0.17 ± 0.48	0.002
h-3, uurelated thoughts		1.00	1.50 ± 0.65	0.50 ± 0.78	0.000
h-4, distracted by sounds		1.00	1.06 ± 0.89	0.29 ± 0.46	0.001
childhood	0.87				
h-1, easily distracted		1.00	1.53 ± 0.73	0.25 ± 0.44	0.000
h-2, easily distracted (others)		1.00	1.31 ± 0.82	0.17 ± 0.38	0.000
h-3, distracted by sounds		1.00	1.03 ± 0.81	0.13 ± 0.44	0.000
h-4, difficulty getting back to as task		0.98	1.42 ± 0.69	0.17 ± 0.38	0.000
i. Forgetful					
adulthood	0.79				
i-1, forgetful		1.00	1.22 ± 0.72	0.21 ± 0.50	0.000
i-2, forgetful (others)		1.00	0.69 ± 0.82	0.08 ± 0.28	0.001
i-3, forgets things in work		1.00	0.49 ± 0.61	0.08 ± 0.28	0.004
i-4, forgetful things in home		1.00	0.94 ± 0.79	0.29 ± 0.62	0.001
childhood	0.85				
i-1, forgetful		1.00	1.89 ± 0.85	0.04 ± 0.20	0.000
i-2, forgetful (others)		1.00	1.00 ± 0.82	0.13 ± 0.33	0.000
i-3, forgets restarting an unfinished task		1.00	0.92 ± 0.73	0.08 ± 0.28	0.000
i-4, forgetful doing daily routine		1.00	1.03 ± 0.87	0.21 ± 0.50	0.001

Calculations of reliability measures (α, κ) and *U*-test were based on participants’ responses to the 72 questions on a 3-points scale (0 = never, 1 = sometimes, 2 = often/always). A response of 2 to one or more of the four questions for each symptom in childhood or adulthood constitutes a positive score for that symptom in that period. Others = other people point out that the respondent has the difficulty. Help from others = another person help the respondent with the difficulty

*ASIA* Assessment System for Individuals with ADHD, *ns* not significant

**Table 2:** Internal consistency reliability, interrater reliability, and discriminant validity for ASIA ADHD criterion A hyperactivity-impulsivity symptoms

Hyperactivity-Impulsivity(9 symptoms: a to i)	α	κ	Mean score ± SD		*p* (*U*-test)
4 questions (1–4 each for adulthood and childhood)			ADHD (*n* = 36)	non-ADHD (*n* = 24)	
a. Fidgeting					
adulthood (age ≥17)	0.83				
a-1, fidgeting		0.98	0.97 ± 0.84	0.25 ± 0.53	0.001
a-2, fidgeting (others)		1.00	0.36 ± 0.63	0.04 ± 0.20	0.020
a-3, fidgeting with one’s hair, taps one’s pencils, etc.		1.00	1.00 ± 0.79	0.25 ± 0.44	0.000
a-4, gets tired when seating		1.00	0.75 ± 0.90	0.04 ± 0.20	0.001
childhood (age < 12)	0.84				
a-1, fidgeting		1.00	1.03 ± 0.91	0.33 ± 0.56	0.003
a-2, fidgeting (others)		1.00	0.67 ± 0.82	0.04 ± 0.20	0.001
a-3, help from others		1.00	0.44 ± 0.77	0.00 ± 0.00	0.005
a-4, fiddles with one’s hair, taps one’s pencil, etc.		1.00	1.22 ± 0.86	0.25 ± 0.53	0.000
b. Leaves seat					
adulthood	0.73				
b-1, leaves seat		1.00	0.14 ± 0.35	0.00 ± 0.00	n.s.
b-2, leaves seat (others)		1.00	0.14 ± 0.42	0.00 ± 0.00	n.s.
b-3, looks for an active task		1.00	0.58 ± 0.73	0.21 ± 0.50	0.027
b-4, sits with extra effort		1.00	0.64 ± 0.79	0.00 ± 0.00	0.000
childhood	0.92				
b-1, leaves seat		1.00	0.31 ± 0.62	0.00 ± 0.00	0.014
b-2, leaves seat (others)		1.00	0.31 ± 0.54	0.00 ± 0.00	0.014
b-3, help from others		1.00	0.22 ± 0.54	0.00 ± 0.00	0.037
b-4, leaves seat in special occasion (e.g. a funeral)		1.00	0.22 ± 0.54	0.00 ± 0.00	0.037
c. Excessive running/climbing					
adulthood	0.75				
c-1, excessive running/restlessness		1.00	0.56 ± 0.73	0.08 ± 0.28	0.004
c-2, too much active (others)		1.00	0.36 ± 0.68	0.08 ± 0.28	n.s.
c-3, feels tired down when seating		1.00	0.83 ± 0.84	0.21 ± 0.50	0.002
c-4, can not help doing something		1.00	0.83 ± 0.81	0.38 ± 0.57	0.027
childhood	0.82				
c-1, excessive running/climbing		1.00	0.69 ± 0.82	0.13 ± 0.33	0.003
c-2, excessive running/climbing (others)		1.00	0.42 ± 0.69	0.08 ± 0.28	0.360
c-3, can not help climbing		1.00	0.50 ± 0.73	0.17 ± 0.38	n.s.
c-4, feels restless when sheeted		1.00	0.81 ± 0.82	0.08 ± 0.28	0.000
d. Difficulty playing quietly					
adulthood	0.86				
d-1, difficulty playing quietly		1.00	0.36 ± 0.63	0.08 ± 0.28	n.s.
d-2, being told “Be quiet”, “Shut up”		1.00	0.33 ± 0.58	0.08 ± 0.28	n.s.
d-3, speak too much loudly and quickly		1.00	0.43 ± 0.69	0.04 ± 0.20	0.010
d-4, can not regulate one’s tone of voice		1.00	0.44 ± 0.60	0.13 ± 033	0.025
childhood	0.85				
d-1, difficulty playing quietly		1.00	0.67 ± 0.79	0.04 ± 0.20	0.000
d-2, being told “Be quiet		1.00	0.58 ± 0.80	0.04 ± 0.28	0.0007
d-3, speak loudly when watching TV		1.00	0.56 ± 0.69	0.21 ± 0.58	0.018
d-4, can not regulate one’s tone of voice		1.00	0.44 ± 0.73	0.04 ± 0.20	0.012
e. On the go					
adulthood	0.78				
e-1, on the go		1.00	0.42 ± 0.73	0.00 ± 0.20	0.005
e-2, on the go (others)		1.00	0.28 ± 0.65	0.04 ± 0.20	n.s.
e-3, can’t slow down		1.00	0.53 ± 0.73	0.21 ± 0.50	n.s.
e-4, feels uncomfortable at a long meeting		1.00	0.64 ± 0.79	0.08 ± 0.28	0.002
childhood	0.85				
e-1, on the go		1.00	0.67 ± 0.79	0.04 ± 0.20	0.000
e-2, on the go (others)		1.00	0.56 ± 0.87	0.08 ± 0.28	0.027
e-3, help from others		1.00	0.25 ± 0.60	0.00 ± 0.00	0.037
e-4, absorbed in something		1.00	0.50 ± 0.69	0.21 ± 0.50	n.s.
f. Talks excessively					
adulthood	0.86				
f-1, talk excessively		0.98	0.81 ± 0.71	0.29 ± 0.55	0.003
f-2, criticized as “born with a big mouth”		1.00	0.47 ± 0.77	0.04 ± 0.20	0.011
f-3, one’s talk bothers family, coworkers or friends		1.00	0.47 ± 0.69	0.08 ± 0.28	0.013
f-4, can not stop talking once started		1.00	0.94 ± 0.82	0.29 ± 0.46	0.002
childhood	0.92				
f-1, talk excessively		1.00	0.69 ± 0.82	0.13 ± 0.44	0.002
f-2, criticized as “shut up”		1.00	0.58 ± 0.84	0.13 ± 0.44	0.016
f-3, help from others		1.00	0.53 ± 0.81	0.04 ± 0.20	0.007
f-4, one’s talk bothers classmates, family or friends		1.00	0.39 ± 0.68	0.00 ± 0.00	0.005
g. Blurts out answers					
adulthood	0.80				
g-1, blurts out answers		1.00	0.56 ± 0.77	0.04 ± 0.20	0.002
g-2, blurts out answers (others)		0.98	0.58 ± 0.80	0.00 ± 0.00	0.001
g-3, slips of the tongue		1.00	0.92 ± 0.73	0.33 ± 0.48	0.002
g-4, rules conversation one-sidedly		1.00	0.58 ± 0.77	0.04 ± 0.20	0.001
childhood	0.85				
g-1, blurts out answers		1.00	0.83 ± 0.81	0.13 ± 0.33	0.000
g-2, blurts out answers (others)		0.98	0.67 ± 0.82	0.00 ± 0.00	0.000
g-3, jerks what comes up in one’s mind		1.00	0.53 ± 0.77	0.17 ± 0.38	n.s.
g-4, slips of the tongue		0.98	0.78 ± 0.83	0.13 ± 0.33	0.001
h. Difficulty awaiting turn					
adulthood	0.64				
h-1, difficulty awaiting turn		1.00	0.25 ± 0.50	0.08 ± 0.28	n.s.
h-2, difficulty awaiting turn (others)		1.00	0.11 ± 0.31	0.00 ± 0.00	n.s.
h-3, difficulty doing something in turn		0.98	0.19 ± 0.52	0.04 ± 0.20	n.s.
h-4, feels frustrated at a red right or traffic jam		0.98	0.83 ± 0.84	0.25 ± 0.44	0.006
childhood	0.74				
h-1, difficulty awaiting turn (others)		1.00	0.53 ± 0.28	0.08 ± 0.73	0.008
h-2, difficulty awaiting turn (others)		1.00	0.36 ± 0.68	0.00 ± 0.00	0.009
h-3, difficulty waiting in activities (e.g. club activities)		1.00	0.14 ± 0.35	0.00 ± 0.00	n.s.
h-4, wants to do things first in the group		1.00	0.19 ± 0.46	0.04 ± 0.20	n.s.
i. Interrupts or intrudes					
adulthood	0.76				
i-1, interrupts or intrudes		1.00	0.17 ± 0.44	0.00 ± 0.00	n.s.
i-2, interrupts or intrudes (others)		1.00	0.22 ± 0.54	0.08 ± 0.28	n.s.
i-3, takes over what others are doing		1.00	0.25 ± 0.52	0.04 ± 0.20	n.s.
i-4, interferes with someone’s work		1.00	0.31 ± 0.23	0.04 ± 0.20	0.021
childhood	0.84				
i-1, interrupts or intrudes		1.00	0.58 ± 0.69	0.00 ± 0.00	0.000
i-2, interrupts or intrudes (others)		1.00	0.53 ± 0.77	0.08 ± 0.28	0.012
i-3, cuts in other’s play without permission		0.98	0.44 ± 0.73	0.08 ± 0.28	0.012
i-4, play with other’s toys without asking		1.00	0.36 ± 0.59	0.00 ± 0.00	0.003

Calculations of reliability measures (α, κ) and *U*-test were based on participants’ responses to the 72 questions on a 3-points scale (0 = never, 1 = sometimes, 2 = often/always). A response of 2 to one or more of the four questions for each symptom in childhood or adulthood constitutes a positive score for that symptom in that period. Others = other people point out that the respondent has the difficulty. Help from others = another person help the respondent with the difficulty

*ASIA* Assessment system for individuals with ADHD, *ns* not significant

For the criteria B, C, D, and E, raw agreement rates between the two raters were 1.00, 1.00, 1.00, and 0.97, respectively.

### Validity

The consensus DSM-5 diagnoses endorsed 35 ASIA ADHD diagnoses and 24 ASIA non-ADHD diagnoses. Regarding ADHD subtypes, the consensus DSM-5 diagnoses endorsed 28 out of the 28 ASIA predominantly inattention presentations and 7 out of the 7 ASIA combined presentations. One case classed as other specified ADHD in the consensus diagnosis was judged as a case of non-ADHD in the ASIA diagnosis.

As shown in Table 1, 71 out of the 72 questions on inattention showed significantly higher ratings in the ADHD group than in the non-ADHD group. The remaining question (e-2 of the adulthood questions) showed higher ratings in the ADHD group than in the non-ADHD group but this difference was not significant.

As shown in Table 2, 54 out of the 72 questions on hyperactivity–impulsivity showed significantly higher ratings in the ADHD group than in the non-ADHD group. ADHD group scores were higher for the remaining 18 questions than in the non-ADHD group but these differences were not significant.

Table 3 shows that the correlation coefficients (*r*) between ASIA ADHD criterion A total and subscale scores in adulthood and childhood and the J-CAARS-S subscale scores ranged from 0.443 to 0.857, except for J-CAARS-S subscale D (ranging from 0.160 to 0.415). In particular, the correlations between scores on the corresponding ASIA ADHD criterion A and the J-CAARS-S subscales (indicated by **bold characters **in Table 3) ranged from 0.657 to 0.857.

**Table 3:** Correlations (*r*) between subscale scores of ASIA ADHD criterion A and J-CAARS-S (n = 60)

	ASIA adult total	AISIA IA adult	ASIA HI adult	ASIA child total	ASIA IA child	ASIA HI child
J-CAARS-S A	0.789**	0.857**	0.510**	0.631**	0.674**	0.443**
J-CAARS-S B	0.700**	0.518**	0.801**	0.634**	0.484**	0.702**
J-CAARS-S C	0.715**	0.642**	0.657**	0.641**	0.579**	0.589**
J-CAARS-S D	0.342**	0.415**	0.160	0.350**	0.351**	0.276**
J-CAARS-S E	0.821**	0.853**	0.586**	0.691**	0.719**	0.510**
J-CAARS-S F	0.677**	0.515**	0.752**	0.627**	0.491**	0.679**
J-CAARS-S G	0.845**	0.776**	0.748**	0.742**	0.684**	0.665**
J-CAARS-S H	0.786**	0.738**	0.674**	0.712**	0.657**	0.638**

*ASIA* Assessment system for individuals with ADHD, *IA* Inattention, *HI* Hyperactive-Impulsive. *J-CAARS-S* Japanese version of Conner’s Adult ADHD Rating Scale-Self Reports, *A* Inattention/Memory problems, *B* Hyperactivity/Restlessness, *C* Impulsivity/Emotional lability, *D* Problems with self-concept, *E* DSM-IV inattentive symptoms, *F* DSM-IV hyperactive-impulsive symptoms, *G* DSM-IV total ADHD symptoms, *H* ADHD index. A framed cell indicates corresponding items between the ASIA and J-CAARS-S subscales

*p <0.05, **p <0.01

## Discussion

ASIA ADHD criterion A showed acceptable internal consistency reliability for the nine inattention and nine hyperactivity–impulsivity symptoms in both adulthood and childhood with the exception of symptom “h” (difficulty waiting turn) for adulthood hyperactivity–impulsivity (α = 0.64). However, there is some disagreement about the acceptable values of α; these range from 0.70 to 0.95. A low value of α can be a result of a small number of questions, poor interrelatedness between items, or heterogeneous constructs [22]. High values of κ on the 144 questions (0.98–1.00) on criterion A (κ between 0.81 and 1.00 is interpreted as almost perfect [23]) and high raw agreement rates (0.97–1.00) for the four questions on criteria B, C, D, and E indicate satisfactory interrater reliability for the ASIA ADHD criteria. These high values of κ and raw agreement rates could partly result from the method of investigating interrater reliability (i.e., the second author reviewed videotaped interviews conducted by the first author during which participants gave their responses). This method was selected to avoid burdening participants with two independent interviews. For the same reason, test–retest reliability was not examined in this study. To ensure the reliability of the ASIA interview, a training program for prospective interviewers should be developed [22, 24].

The consensus diagnoses agreed with the ASIA ADHD diagnoses (ADHD vs. non-ADHD and subtype diagnoses) in 59 of the 60 cases, indicating acceptable concurrent validity of the ASIA ADHD criteria. In one case, the consensus diagnosis was Other Specified ADHD, whereas the ASIA diagnosis was non-ADHD. This case met the other specified ASIA ADHD diagnosis except for criteria A and D. In the ASIA interview, this case had three positive inattention symptoms and four positive hyperactivity–impulsivity symptoms in childhood, and three positive inattention symptoms and three positive hyperactivity–impulsivity symptoms in adulthood, along with a Global Assessment of Functioning score of 72. The disagreement in diagnoses for this case supports Diller’s point that “although severe and even moderate ADHD might not be too difficult to diagnose, mild ADHD, especially the inattentive type, is wide open to interpretation as to what behavior crosses the line from a variation of normal to the deviancy of disorder [25].” The diagnosis of mild cases of ADHD should be holistically made and refer to extensive information, including ASIA and other available data.

The correlations between ASIA ADHD criterion A subscale scores and J-CAARS-S subscale scores were moderate to high (*r* = 0.44–0.86) and the correlations for the corresponding subscale scores were particularly high (*r* = 0.66–0.86). We considered the relatively low correlations between ASIA ADHD criterion A scores and the J-CAARS-S subscale D scores reasonable because the subscale D measures problems of self-concept that have no direct relationship with ADHD symptoms. In a similar comparison, correlations between corresponding items on the CAADID DSM-IV ADHD symptom counts and the Conners’ adult ADHD rating scale-Self Report (*n* = 30) ranged from 0.20 to 0.52 [26]. Overall, our findings indicate acceptable concurrent validity for ASIA ADHD criterion A.

The ADHD group scored significantly higher on 125 of the 144 questions of ASIA ADHD criterion A than the non-ADHD group, indicating acceptable discriminant validity. The remaining 19 questions (1 for inattention and 18 for hyperactivity–impulsivity) failed to show a significant difference between the two groups. This is partly explained by small statistical power resulting from the relatively small number of participants, especially for ADHD with combined presentation. The small number of ADHD combined presentation cases might partly be a result of the attenuating nature of hyperactivity–impulsivity symptoms in adulthood, as described in the DSM-5 ADHD criteria for hyperactivity symptom c, which indicates that “excessive running/climbing may be limited to feeling restless in adolescents or adults [4].” However, because the scores were higher (though not significantly so) in the ADHD group than in the non-ADHD group for these 19 questions, the ASIA will retain these questions for further research. Furthermore, as mentioned in the Methods, we consider that the process of creating the 144 questions ensures that ASIA ADHD criterion A has sufficient content validity.

For a diagnosis of Other Specified ADHD, we set an operational rule that four symptoms in adulthood and three to five symptoms in childhood of either inattention or hyperactivity-impulsivity should be present (as shown in Fig. 1) with reference to Surman et al.’s operationalized rule for DSM-IV ADHD-Not Otherwise Specified (NOS; at least five but less than six out of nine symptoms for inattention or hyperactivity/impulsivity) [27]. These numbers might be too conservative. However, excessive expansion of the range of other specified ADHD would run the risk of increasing false-positive rates. Faraone et al. [28] found little evidence for the validity of subthreshold ADHD (having three, four, or five inattention or hyperactivity/impulsivity symptoms in DSM-IV) judging from the clinical features of probands and the pattern of transmission of ADHD among relatives. A further study is needed to confirm the legitimacy of our thresholds for the number of ADHD symptoms.

There are several differences between existing semi-structured interviews based on DSM-IV and the ASIA. CAADID and DIVA employ dichotomous responses (i.e., present or absent), whereas AISRS employs a 4-point scale. We used a 3-point scale for the ASIA ADHD criterion A questions for the following reasons: to increase the precision of the severity rating of symptoms compared to a 2-point scale, to reduce the number of response choices for Japanese participants who are unfamiliar with frequency responses, and to shed light on subthreshold ADHD (i.e., cases not diagnosed as ADHD or Other Specified ADHD but having certain ADHD symptoms) for future research. In the CAADID and DIVA techniques, interviewees are prompted (if necessary) by examples of each symptom, the numbers of which differ from symptom to symptom. For every ASIA ADHD criterion A symptom, the first question (which is most faithful to the DSM-5 symptom description) is followed by three example questions. This unification enables clinicians or researchers to easily measure several psychometric properties.

Comorbidity of pervasive developmental disorders (ASD and Rett’s disorder) and ADHD is not permitted in DSM-IV but permitted in DSM-5. There were only three participants in this study diagnosed with both ASD and ADHD. Determining whether there is any symptomatic difference between individuals with ADHD only and those with both ADHD and ASD would be of great clinical interest and should be investigated in a further study.

There are some limitations to our study. In the interrater reliability study, the second author rated participants while watching videotaped interviews by the first author instead of directly administering the ASIA, because it would have been time-consuming to administer another assessment. The ratings of the second author, who was blind to any participant information except for her ASIA ratings, were used to diagnose ADHD. However, as the second author rated the interview while watching the videotape of the first author’s interviews, she may have been influenced by the first author’s biases, even though all possible measures were taken to make the first author blind to any information about participants except for the ASIA ratings. This shortcoming needs to be addressed by a further study employing a more rigorous methodology (i.e., two interviewers independently conducting ASIA interviews). Even with the inherent methodological problems, it was important to examine interrater reliability in the present study, as this semi-structured diagnostic interview can be administered by a variety of clinicians. Similarly, when confirming concurrent validity, the third and the fourth authors made diagnoses by referring to detailed clinical records of the cases instead of using face-to-face clinical interviews with potential ADHD patients. Although this lessened the participants’ burden, this simplified procedure may have allowed bias in the diagnosis of ADHD in adults. This shortcoming needs to be resolved in a future study.

## Conclusions

The ASIA ADHD criteria showed acceptable internal consistency reliability; interrater reliability; and concurrent, discriminant, and content validity and could be useful in clinical practice and research. However, further investigation of this instrument is necessary.

## Additional file

Additional file 1:
**Sample questions of ASIA ADHD criterion A.** Eight sample questions of ASIA ADHD criterion A illustrating what questions in ASIA are like.

## Abbreviations

ADHD: Attention-deficit/hyperactivity disorder
ASIA: Assessment System for Individuals with ADHD
CAADID: Conners’ Adult ADHD Diagnostic Interview for ADHD in Adults
DIVA: Diagnostisch Interview voor ADHD
J-CAARS-S: Japanese version of Conners’ adult ADHD rating scales-Self Report
