# Supplementary material for: Reliability and validity of ADHD diagnostic criteria in the Assessment System for Individuals with ADHD (ASIA): a Japanese semi-structured diagnostic interview
Source: BMC Psychiatry. 2015 Jun 20;15:130. doi: 10.1186/s12888-015-0525-7 (PMC4474425; doi:10.1186/s12888-015-0525-7)
Supplement: Additional file 1: — Sample questions of ASIA ADHD criterion A. Eight sample questions of ASIA ADHD criterion A illustrating what questions in ASIA are like. [file 12888_2015_525_MOESM1_ESM.docx]

**Additional file 1**

**Sample questions of ASIA ADHD criterion A**

1. **Inattention symptom b (difficulty sustaining attention) on ASIA ADHD criterion A (b-3 and b-4 for adulthood and childhood)**

(b-3 for adulthood)

When you are at work or school in the daytime, how often do you experience being in a daze, feeling “spaced-out,” yawning during activities, or being unable to focus on your task, even though you slept well the previous night?

never □ sometimes □ often/always (often or more) □

(b-4 for adulthood)

Most people have a tendency to focus on what they are interested in and to avoid focusing on what they are not so interested in. How often do you experience a big difference in concentration between what you are interested in and what you are not so interested in, and feel you cannot concentrate on the things people expect you to focus on, or on the things you are not so interested in?

never □ sometimes □ often/always (often or more) □

(b-3 for childhood)

When you were at school or home in the daytime, how often did you experience being in a daze, feeling “spaced-out” in a class, inability to focus on your lessons, or placing your head on the desk, even though you had slept well the previous night? (Cases where the lessons were too challenging for you to understand, making you feel bored, are not applicable).

never □ sometimes □ often/always (often or more) □

(b-4 for childhood)

Most people have a tendency to focus on what they are interested in and avoid focusing on what they are not so interested in, especially in childhood. How often did you experience, when you were a child, a big difference in concentration between what you were interested in and what you were not so interested in, and that you could not concentrate on the things your teachers or parents expected you to focus on, or on the things you were not so interested in?

never □ sometimes □ often/always (often or more) □

1. **Hyperactivity-impulsivity symptom b (difficulty sustaining attention) on ASIA ADHD criterion A (b-3 and b-4 for adulthood and childhood)**

(b-3 for adulthood)

Question: How often have you looked for more active work because you would like to move around rather than be seated?

never □ sometimes □ often/always □

(b-4 for adulthood)

Question: How often have you needed great effort to remain seated at a meeting or in other similar situations?

never □ sometimes □ often/always □

(b-3 for childhood)

Question: How often did you receive special treatment in class, like being allowed to walk around, move your body, or go out of the class because you could not sit still?

never □ sometimes □ often/always □

(b-4 for childhood)

Question: How often did you fail to remain seated when you were eating, doing your homework, or attending a funeral or other solemn occasions? (This depends largely on your age, so please try to compare your behavior with that of your classmates at the time, as far as you can remember it).

never □ sometimes □ often/always □
